# Targeting the extradomain A of fibronectin allows identification of vascular resistance to antiangiogenic therapy in experimental glioma

**DOI:** 10.18632/oncotarget.25570

**Published:** 2018-06-12

**Authors:** Güliz Acker, Sophie Käthe Piper, Anna Lena Datwyler, Thomas Broggini, Irina Kremenetskaia, Melina Nieminen-Kelhä, Janet Lips, Ulrike Harms, Susanne Mueller, Gilla Lättig-Tünnemann, Eveline Trachsel, Alessandro Palumbo, Dario Neri, Jan Klohs, Matthias Endres, Peter Vajkoczy, Christoph Harms, Marcus Czabanka

**Affiliations:** ^1^ Charité-Universitätsmedizin Berlin, Corporate Member of Freie Universität Berlin, Humboldt-Universität zu Berlin, and Berlin Institute of Health, Department of Neurosurgery, Berlin, Germany; ^2^ Charité-Universitätsmedizin Berlin, Corporate Member of Freie Universität Berlin, Humboldt-Universität zu Berlin, and Berlin Institute of Health, Center for Stroke Research, Berlin, Germany; ^3^ Charité-Universitätsmedizin Berlin, Corporate Member of Freie Universität Berlin, Humboldt-Universität zu Berlin, and Berlin Institute of Health, Institute of Biometry and Clinical Epidemiology, Berlin, Germany; ^4^ Charité-Universitätsmedizin Berlin, Corporate Member of Freie Universität Berlin, Humboldt-Universität zu Berlin, and Berlin Institute of Health, Department of Neurology and Experimental Neurology, Berlin, Germany; ^5^ Department of Chemistry and Applied Biosciences, Institute of Pharmaceutical Sciences, ETH Zurich, Zurich, Switzerland; ^6^ Institute for Biomedical Engineering, University of Zurich and ETH Zurich, Zurich, Switzerland

**Keywords:** NIRF imaging, antiangiogenic resistance, F8, glioma, SF126

## Abstract

**Introduction:**

Clinical application of antiangiogenic therapy lacks direct visualization of therapy efficacy and vascular resistance. We aimed to establish molecular imaging during treatment with sunitinib using the fibronectin extradomain A specific small immunoprotein(SIP)-F8 in glioma.

**Methods:**

Biodistribution analysis of F8-SIP-Alexa-555 was performed in SF126-glioma bearing or control mice (*n* = 23 and 7, respectively). Intravital microscopy(IVM) was performed on a microvascular level after 7 days (*n* = 5 per group) and subsequently after 6 days of sunitinib treatment (*n* = 4) or without (*n* = 2).

Additionally, near infrared fluorescence(NIRF) imaging was established with F8-SIP-Alexa-750 allowing non-invasive imaging with and without antiangiogenic treatment in orthotopic tumors (*n* = 38 divided in 4 groups). MRI was used to determine tumor size and served as a reference for NIRF imaging.

**Results:**

F8-SIP demonstrated a time and hemodynamic dependent tumor specific accumulation. A significantly higher vascular accumulation occurred with antiangiogenic treatment compared to untreated tumors enabling visualization of resistant tumor vessels by F8-SIP-mediated NIRF imaging. In orthotopic tumors, sunitinib reduced F8-SIP-Alexa-750 enrichment volume but not fluorescence intensity indicative of F8-SIP accumulation in fewer vessels.

**Conclusion:**

F8-SIP is highly tumor specific with time and hemodynamic dependent biodistribution. The higher vascular accumulation to remaining vessels enables molecular imaging and targeting of therapy resistant tumor vessels.

## INTRODUCTION

Clinical treatment of malignant glioma is currently being revolutionized by an improved understanding of the molecular profile of the disease leading not only to a novel classification of gliomas but also to novel treatment algorithms [1]. A molecular sub classification of glioblastoma multiforme (GBM) has been proposed that allows differentiation between four GBM subtypes [2]. The proneural GBM subtype has been postulated as the “proangiogenic” subtype [2] and the AVAGlio trial showed a significantly increased overall survival if the VEGF antibody bevacizumab was applied in addition to radiation therapy and temozolomide chemotherapy [3]. The other three subgroups did not benefit from antiangiogenic treatment in overall survival probably due to so far unknown vascular resistance mechanisms [3]. These findings highlight the importance of early identifying patients that are suitable for antiangiogenic treatment and respond to the treatment. Antibody based vascular targeting strategies allow specific delivery of therapeutic or diagnostic agents directly to the vascular system of tumors [4]. The Extra-domain A (EDA) of fibronectin is one of the most appealing neovascular target molecules in highly-vascularized tumors like malignant glioma [5]. The small immunoprotein (SIP) fragment F8 (F8-SIP) of EDA based vascular targeting enables molecular imaging of the vascular system. This potentially allows to identify patients that benefit from antiangiogenic treatment and to visualize therapy-resistant tumor areas. The aim of our study was to characterize microvascular binding characteristics of F8-SIP by intravital microscopy and to assess its use for non-invasive imaging of orthotopic glioma angiogenesis by near infrared fluorescence (NIRF) imaging under antiangiogenic treatment.

## RESULTS

### Microvascular bio-distribution of F8-SIP

**Figure 1:**
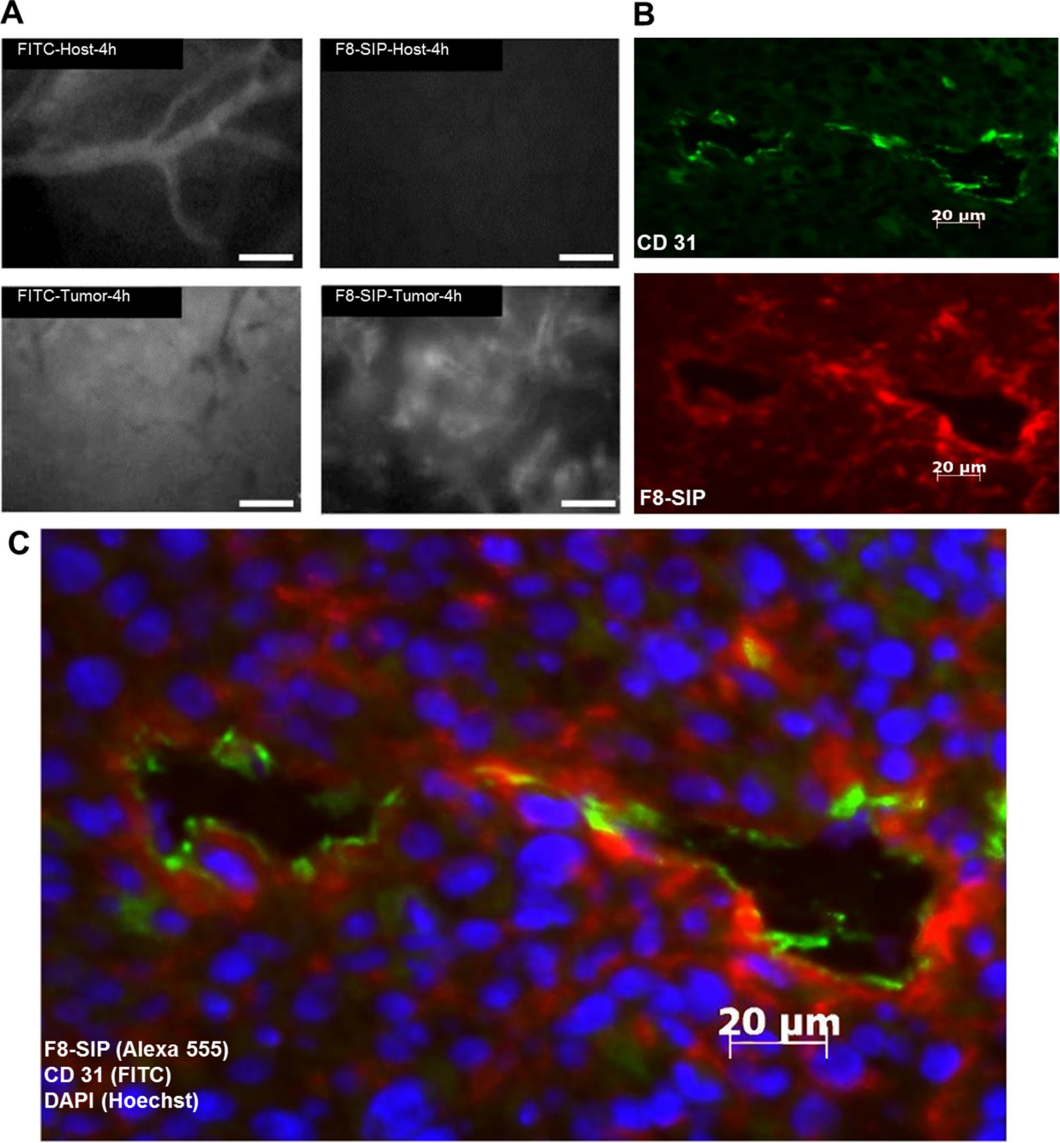
Bio-distribution of F8-SIP-Alexa555 in SF126 skin chamber tumors (**A**) Mice with skin chambers received 30000 SF126 cells 2 days after surgery or control injection (5 animals per group). After tumor growth of further 7 days, F8-SIP-Alexa-555 antibody and FITC was injected. Representative demonstration of vascular accumulation of F8-SIP around tumor vessels using intravital fluorescence video microscopy (IFVM). No antibody accumulation could be detected around host tissue blood vessels 4 h after injection. Application of FITC demonstrated very leaky tumor blood vessels in comparison to host vessels. Scale bar represents 20 µm. (**B**) Animals were sacrificed after 24 h and tissue was further processed for immunohistochemistry (for details see methods section). Endothelial cell staining with CD31 (green) and F8-SIP-Alexa-555 (red) co-localized to the tumor endothelium (ab-luminal accumulation) and extravasation of the antibody into the interstitial space of the tumor is shown. (**C**) Merged CD31 (green) and F8-SIP (red) of B) with DAPI staining (blue) showing tumor and host cell nuclei in higher magnification. Scale bar represents 20 µm.

**Figure 2:**
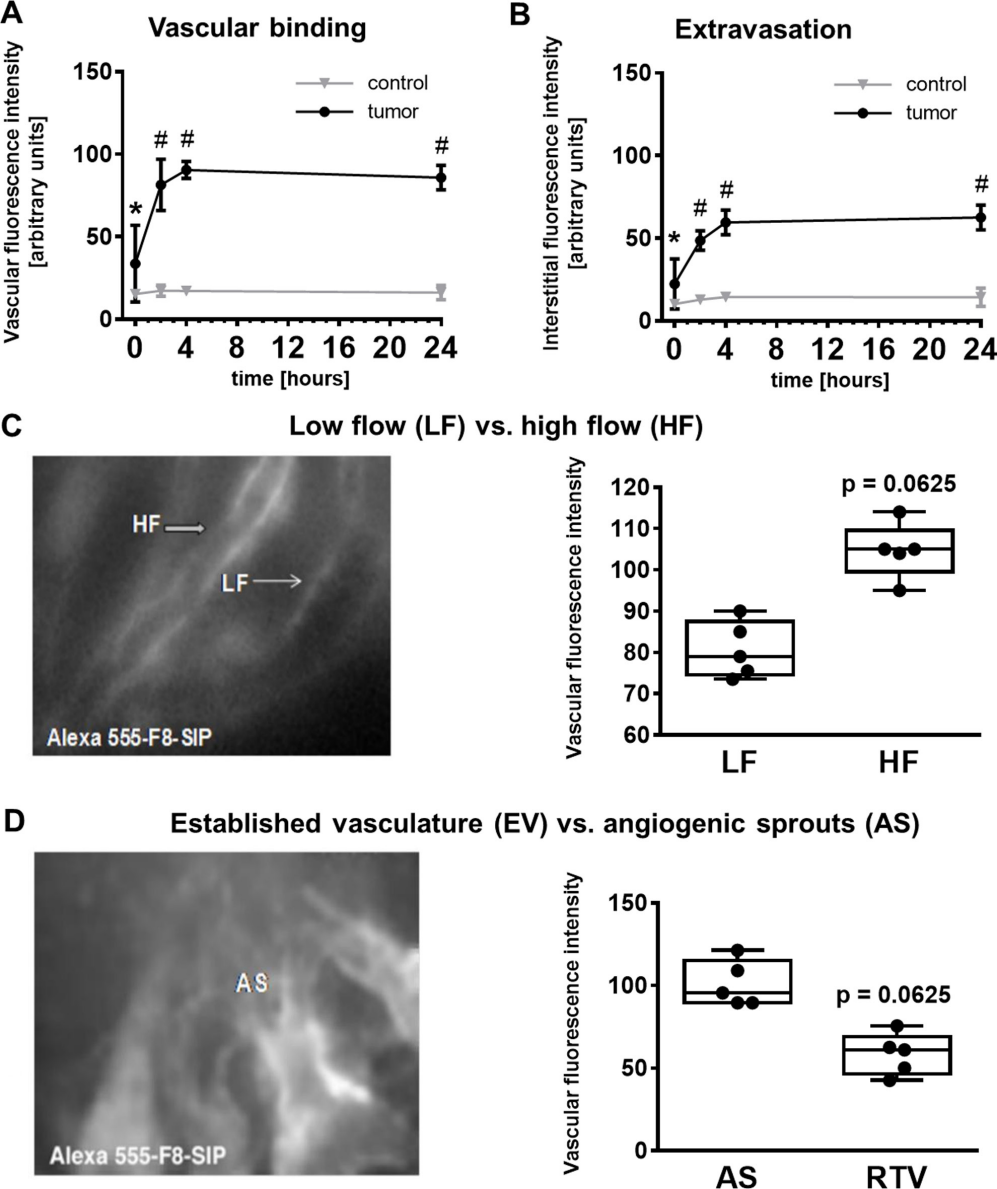
F8-SIP antibody binds to high flow tumor vasculature and angiogenic sprouts (**A**) Intravital microscopy after 7 days of tumor inoculation confirms F8-SIP antibody binding to tumor vasculature as early as 2 h post i.v. injection and stable binding profile until 24 h past the experiment. Data represents mean ± 95% confidence interval, *n* = 5 animals per group, two way repeated measures (RM) ANOVA with Sidak’s posthoc test and F (3, 24) = 31.35, *p* < 0.001 for interaction of tumor and time after injection (tumor accounts for 71.3% of variance, time for 12.4% of variance and interaction of tumor and time for 11% of the variance). Asterisk (^#^) marks *p* < 0.0001 versus control, pound (^*^) marks *p* < 0.0072 versus control. (**B**) Similar to the vascular binding, the F8-SIP antibody extravasates 2 h post i.v. injection and remains bound until the end of the experiment. Data represents mean ± 95% confidence interval, *n* = 5 animals per group, two way RM ANOVA with Sidak’s posthoc test and F (3, 24) = 34.77 and *p* < 0.0001 for interaction of tumor and time after injection; asterisk (^#^) marks *p* < 0.0001 versus control, pound (^*^) marks *p* < 0.05 versus control (tumor accounts for 66.9% of variance, time for 16.5% of variance and interaction of tumor and time for 10.7% of the variance). (**C**) Comparison of low flow (blood flow rate < 20 nl/s) and high flow (blood flow rate > 60 nl/s) tumor vessels revealed a higher F8-SIP binding to high flow tumor vessels (*p* = 0.0625). Data points represent the average of analyzed vessels per animal, *n* = 5 animals, paired Wilcoxon test with 5 pairs. Further analysis of the F8-SIP antibody binding affinity revealed a higher binding in angiogenic sprouts (AS) compared to the established tumor vasculature (EV). Data points represent the average of analyzed vessels per animal, *n* = 5 animals, paired Wilcoxon test with 5 pairs (*p* = 0.0625). (**D**) Boxplots represent median as well as lower and upper limit of the interquartile range (25th and 75th percentile), with whiskers giving the minimum and maximum values.

Three out of 8 mice in the glioma group died during anesthesia and none in the control group resulting in *n* = 5 per group. F8-SIP-Alexa-555 predominantly accumulated in the perivascular space of the tumor vasculature (Figure 1A–1C). Lower F8-SIP-Alexa-555 signals were also found in close proximity to CD31 positive vessels interstitially (Figure 1A–1C). IVFM demonstrated specific accumulation of F8-SIP-Alexa-555 in tumor vasculature after 4 h (mean ± 95% confidence interval): SF126-vasculature: 90.4 ± 3.6 arbitrary units (a.u.); control-vasculature: 17.2 ± 1.2 a.u. (Figures 1B, 2A). Furthermore, extravasation of F8-SIP-Alexa-555 was visualized in tumor interstitium specifically (after 4 h: SF126: 59.5 ± 5.3 a.u.; control: 14.4 ± 1.0 a.u.; Figure 2B), whereas no accumulation or extravasation was detected in the host vasculature. These extravasation and accumulation processes mainly occurred within the first 2 h after F8-SIP-Alexa-555 injection reaching maximum fluorescence intensity in the vasculature 4 h after injection or 24 h in the interstitial space (Figure 2A–2B). Vascular accumulation of F8-SIP-Alexa-555 exhibits strong vascular binding affinity with fluorescence signals remaining roughly constant between 4 and 24 h past intravenous injection (Figure 2A–2B).

Interestingly, microvascular biodistribution was found to be dependent on hemodynamics. In high flow blood vessel (blood flow rate > 60 nl/s) a higher vascular accumulation of F8-SIP-Alexa-555 was observed by IVFM compared to low-flow vessels (blood flow rate < 20 nl/s) with a median fluorescence intensity (given with limits of the interquartile range [IQR]): 105 [99.5 109.5] a.u. vs. 79 [74.5 87.5] a.u. for high and low flow, respectively (Figure 2C, two-tailed Wilcoxon test with 5 pairs, *p* = 0.0625). Moreover, a higher accumulation of F8-SIP-Alexa-555 was evaluated in highly angiogenic sprouts (AS) compared to fully established tumor vessels (RTV) with a median fluorescence intensity in AS of 95.5 [89.5 115.2] a.u. and in RTV of 61 [46.3 69.0] a.u., respectively (Figure 2D, two-tailed Wilcoxon test with 5 pairs, *p* = 0.0625).

### Microvascular consequences of antiangiogenic treatment

**Figure 3:**
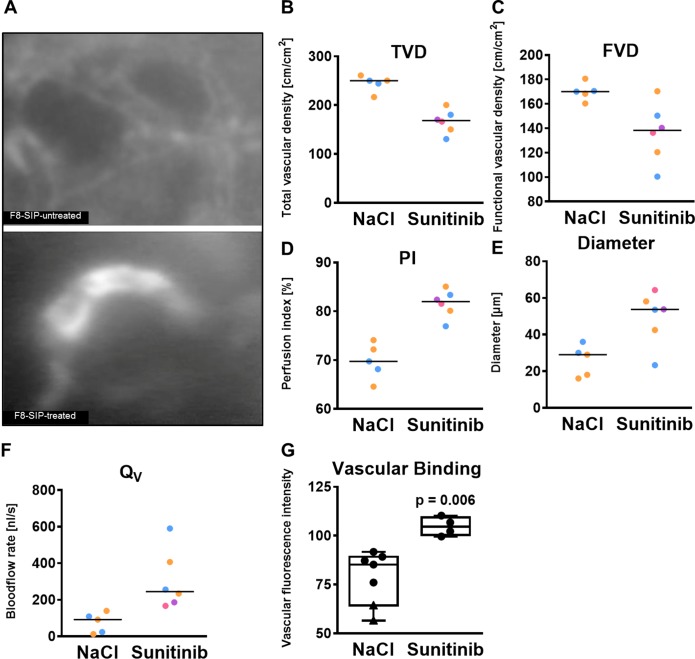
F8-SIP demonstrates improved vascular accumulation in sunitinib resistant tumor vessels (**A**) Mice with skin chambers received 20000 SF126 cells 2 days after surgery or control injection. After tumor growth of further 7 days, treatment groups were randomized for sunitinib treatment (40 mg/kg BW on 6 consecutive days; 4 animals) or control injections with NaCl (2 animals). Representative intravital microscopy images demonstrating an enhanced binding of F8-SIP to tumor vessels after sunitinib treatment compared to untreated tumor vessels (NaCl) after 24 h. (**B**) Total vascular density (TVD) was lower in sunitinib treated tumors compared to untreated tumors. Data points represent *n* = 2 animals each with 2 and 3 tumor fields, respectively, coded by colors in the NaCl-group and *n* = 4 animals with 1 or 2 tumor fields coded by colors in the sunitinib group. Lines indicate the median per group. This data set is a conformation of previously published data and limited due to the availability of antibody [8, 12, 13, 15, 19]. No statistical test was performed. (**C**) Functional vascular density (FVD) was lower after sunitinib therapy. Data points represent *n* = 2 animals each with 2 and 3 tumor fields, respectively, coded by colors in the NaCl-group and *n* = 4 animals with 1 or 2 tumor fields coded by colors in the sunitinib group. Lines indicate the median per group. (**D**) Perfusion index (PI) was higher in sunitinib treated vessel compared to no treatment due to the lower TVD and FVD after sunitinib treatment. Data points represent *n* = 2 animals with 2 and 3 tumor fields, respectively, coded by colors in the NaCl-group and *n* = 4 animals with 1 or 2 tumor fields coded by colors in the sunitinib group. Lines indicate the median per group. (**E**) Mean vascular diameter per region of interest was significantly larger with antiangiogenic therapy. Data points represent *n* = 2 animals each with 2 and 3 tumor fields, respectively, coded by colors in the NaCl-group and *n* = 4 animals with 1 or 2 tumor fields coded by colors in the sunitinib group. Lines indicate the median per group. (**F**) Analysis of microvascular blood flow shows higher blood flow in antiangiogenic therapy resistant blood vessels. Data points represent *n* = 2 animals with 2 and 3 tumor fields, respectively, coded by colors in the NaCl-group and *n* = 4 animals with 1 or 2 tumor fields coded by colors in the sunitinib group. Lines indicate the median per group. (**G**) Vascular fluorescence analysis of NaCl treated animals (circles indicate 7 days of tumor growth (*n* = 5) and triangle indicates 14 days of tumor growth, *n* = 2) and Sun (*n* = 4) treated animals revealed a significantly higher fluorescence signal in sunitinib group. Data points represent averaged values over 5-6 tumor fields per animal, Mann-Whitney *U* test, *p* = 0.006.

Three animals died during or after the treatment period (one sudden death, one early therapy termination, one narcosis incident) and 6 animals finished treatment over 6 days. Additional two animals were excluded for technical reasons (diminished DSC quality) resulting in *n* = 4 animals with sunitinib treatment and *n* = 5 for 7 days and *n* = 2 for 14 days without treatment, respectively (Figure 3). Sunitinib treatment resulted in a lower total vessel density compared to untreated tumors and, though to a smaller extent, less functional vessel density (Figure 3A–3C). This antiangiogenic effect was paralleled by functional alterations with a higher median perfusion index of 82% in therapy resistant tumor blood vessels compared to untreated controls 69%, Figure 3D). Sunitinib resistant tumor blood vessels were characterized by a larger median diameter (53.7 µm versus 25 µm) and higher blood flow rate (245 nl/s versus 91.5 nl/s) compared to untreated tumor vessels (Figure 3E and 3F). In these sunitinib - resistant blood vessels we observed a higher vascular accumulation of F8-SIP-Alexa-555 (median fluorescence after 24 h: 104.5 [100.3 109.4] a.u. treated versus 85.2 [64.3 89.2] a.u. untreated; Mann-Whitney *U* test, *p* = 0.006; Figure 3G). Microvascular permeability was not altered by treatment (mean (SD): 0.91 (0.1) a.u. and 0.91 (0.01), for Sunitinib and NaCl-treated group, respectively).

### Sunitinib treatment results in less angiogenesis and orthotopic glioma growth while inducing vascular resistance if applied in a high but fractionated dosage

In our second set of experiments, an orthotopic tumor mouse model was used and we inoculated SF126 cells from lateral to avoid interference of the scar when performing NIRF imaging of tumor angiogenesis from above the animals.

**Figure 4:**
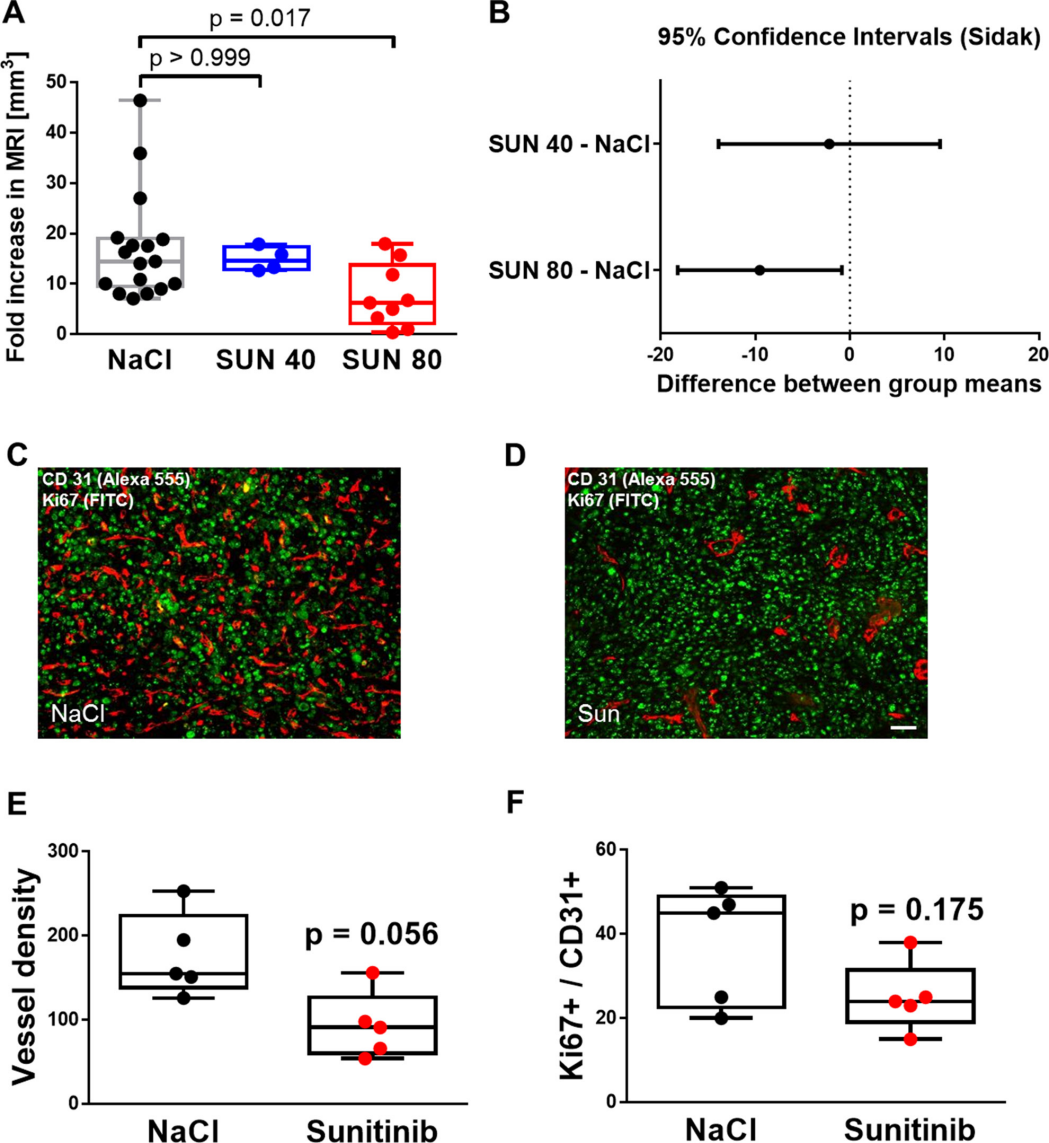
Sunitinib treatment successfully reduces angiogenesis and tumor growth in orthotopic glioma while inducing vascular resistance (**A**) MRI imaging on day 4 and day 9 after lateral inoculation of 20000 SF126 cells resulted in an 14.5 [9.5; 19.0] median and 25th and 75th percentile of IQR fold or 68.3 mm^3^ increase in tumor volume that was reduced to 14.6 [12.8; 17.4] fold or 60.1 mm^3^ (40 mg/ kg BW sunitinib on 5 consecutive days) and 6.3 [2.2; 13.8] median and 25th and 75th percentile of IQR or 30.7 mm^3^ if treated twice with 80 mg/ kg BW on day 4 and 7. Kruskal-Wallis ANOVA with Dunn’s multiple comparison method. (**B**) Plots of the difference between group means of fold decreases of tumor volumes and 95% confidence intervals versus NaCl treated tumors. Only mice treated with 80 mg/ kg BW on day 4 and 7 showed a reduction of approximately 49% of tumor volume on average. One way ANOVA followed by Dunn’s posthoc measurements, *p* = 0.017. (**C** and **D**) Representative images of immunohistochemical analysis of placebo (NaCl) and sunitinib therapy SF126 intracranial gliomas. Tumor vessel proliferation was detected using CD31 endothelial marker (red) and Ki67 proliferation marker (green). Bar indicates 20 µm. (**E**) Quantified results of immunohistochemical analysis for total vascular density by CD31 staining demonstrating lower vascular density in sunitinib compared to NaCl treated tumors; Data points represent averaged values over 5-6 tumor fields per animal, *n* = 5 animals per group, Mann-Whitney *U* test, *p* = 0.056. (**F**) Graphical illustration of quantified results of immunohistochemical analysis for endothelial cell proliferation. Double staining of blood vessels (CD31) and proliferative cells (Ki67) revealed a lower but not statistically significant median count of proliferative tumor endothelial cells with sunitinib treatment compared to placebo (NaCl). Data points represent averaged values over 5-6 tumor fields per animal, *n* = 5 animals per group, Mann-Whitney *U* test, *p* = 0.175.

First, we tested whether sunitinib effectively reduces tumor volume after 5 days of treatment. A daily dose of 80 mg/kg BW was not tolerated well by mice: 3 out of 4 mice died before the second MRI scan on day 9. A dose of 40 mg, if applied daily, did not significantly affect tumor volume (Figure 4A–4B) and 0 of 4 animals died in this group. We then reduced the number of intraperitoneal injections with 80 mg/kg BW from 5 to 2 (day 4 and day 7 after inoculation), which showed a lower mortality (3 out of 12 mice). NaCl treatment was well tolerated with only 1 out of 17 mice dying after the second MRI scan, thus data are still included. The treatment regime of 80 mg/kg BW given twice was kept for all following experiments and showed a lower orthotopic glioma growth rate in the second MRI scan compared to NaCl treatment (median and limits of the IQR): 6.3 [2.2 13.8] fold increase in MRI volume for Sunitinib treated group and 14.5 [9.5 19.0] fold increase in the NaCl treated group (Kruskal-Wallis ANOVA on ranks: *p* = 0.023 and Dunn’s multiple comparison method for pairwise comparison: *p* = 0.017, Figure 4A).

The lower vascular density of subcutaneous SF126 tumors was verified also histologically in orthotopic SF126 tumors. Sunitinib therapy (when applied at 80 mg/kg BW on two non-consecutive days) resulted in a lower vascular density (Figure 4C–4F, 91 [60 127] versus 155 [138.5 224], Mann-Whitney *U* test: *p* = 0.056). Double staining of blood vessels (CD31) and proliferative cells (Ki67) revealed a lower median count of proliferative tumor endothelial cells (24 [19 31.5] versus 45 [22.5 49], Figure 4C–4F), though it did not reach statistical significance (Mann-Whitney *U* test: *p* = 0.175).

### Non-invasive molecular imaging of glioma vasculature using F8-SIP-Alexa-750

**Figure 5:**
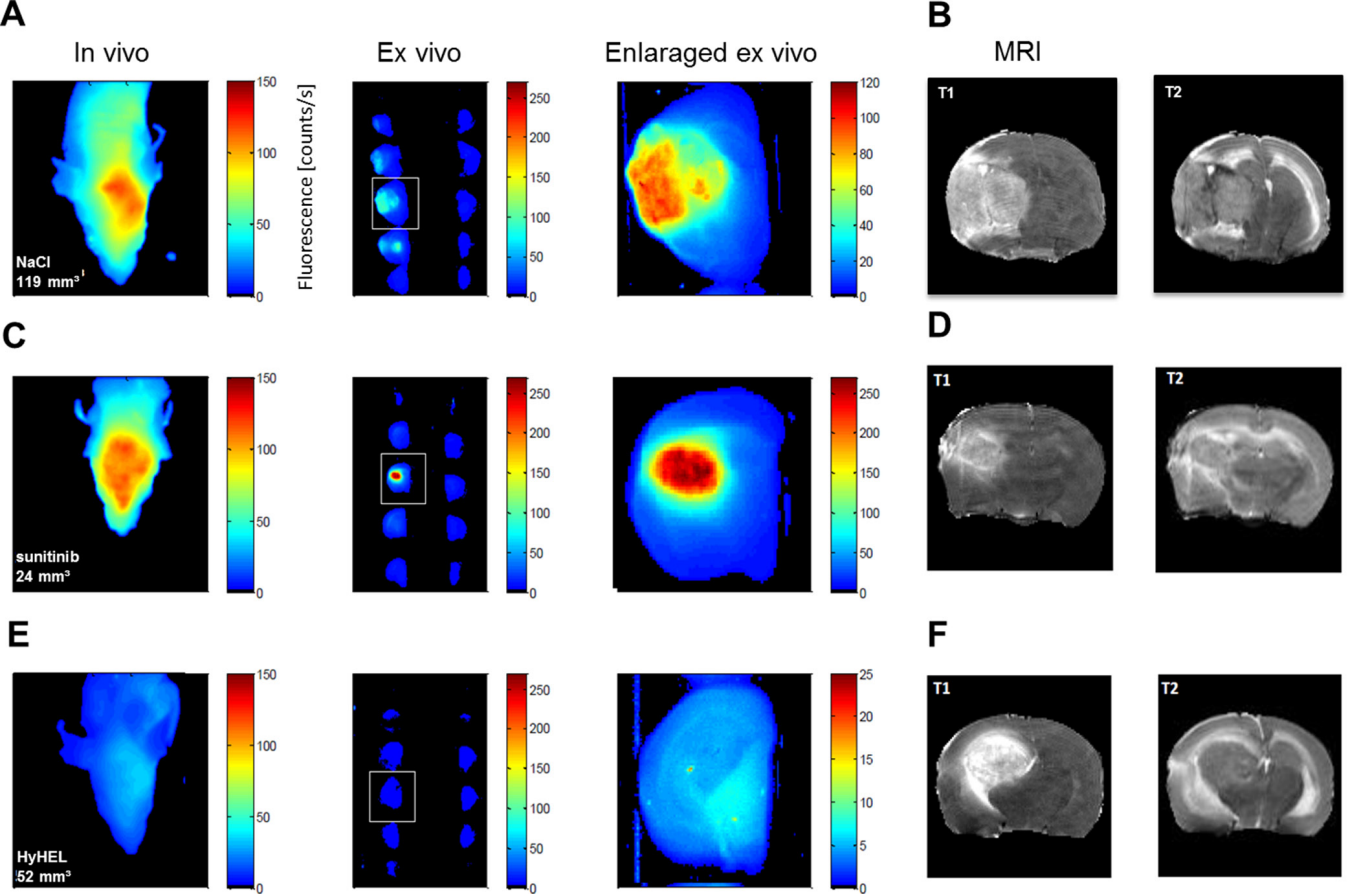
NIRF imaging allows non-invasive molecular imaging of glioma microcirculation (**A**–**F**) Representative noninvasive near infrared fluorescence imaging (NIRF) using F8-SIP-Alexa-750 and magnet resonance imaging (MRI) of NaCl (A, B), 80 mg/ kg BW sunitinib, day 4 & 7 (C, D) or NaCl treated tumors with HyHEL-Alexa-750 controls (E, F) and *ex vivo*-NIRF imaging of 2 mm-thick consecutive brain slices and their respective enlargement (see indicated squares in the brain slices). All *in vivo* and *ex vivo* NIRF images are false colored and normalized to the respected fluorescence counts/s as indicated by the color scale bar except for the enlargements that were individually adapted for better visualization of the bio-distribution.

**Figure 6:**
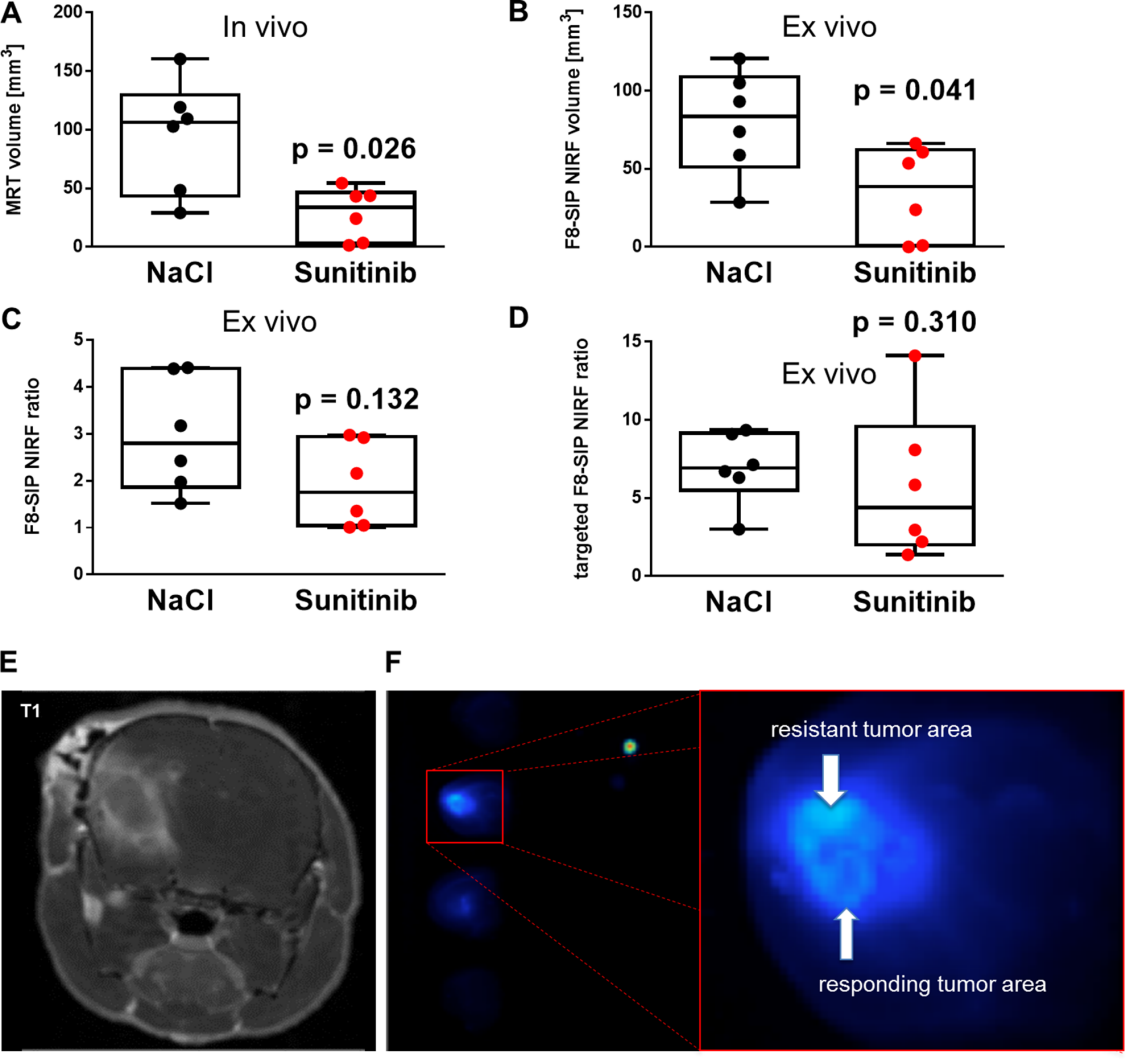
NIRF imaging allows differentiation between therapy resistant and therapy responsive areas in experimental glioma (**A**) Individual glioma volumes measured by MRI. Sunitinib therapy applied on day 4 & 7 resulted in significantly smaller tumor volumes compared to NaCl control treatment (*n* = 6 animals per group, Mann-Whitney *U* test, *p* = 0.026. (**B**) Individual glioma volumes measured by NIRF imaging. Sunitinib therapy resulted in significantly lower NIRF volume based on the F8-SIP antibody distribution (see methods and Supplementary Figure 1B). Mann-Whitney *U* test, *p* = 0.041. (**C**) Fluorescence ratios of F8-SIP-Alexa-750 antibodies showed an enrichment of fluorescent F8-SIP on the ipsilateral SF126 bearing hemispheres compared to contralateral side in both NaCl (median and [IQR]: 2.8 fold [1.9 4.4]) and sunitinib (1.8 fold [1.0 2.9]) treated groups (Mann-Whitney *U* test, *p* = 0.132). (**D**) Targeted fluorescence ratios of F8-SIP-Alexa-750 antibodies within the angiogenic area of slices (see Supplementary Figure 1B) and the mirrored area of the contra-tumoral hemisphere showed an enrichment of fluorescence for both NaCl and sunitinib that did not statistically differ between groups (*p* = 0.310). (**E**) Contrast-enhanced T_1_-weighted MR image of an orthotopically implanted glioma, which is analyzed by NIRF imaging (**F**) demonstrating different treatment responses within the tumor area showing high fluorescence signal in therapy resistant tumor area right next to very low fluorescence signal (sufficient treatment response).

Non-invasive, *in vivo* NIRF imaging of the head and post mortem NIRF imaging of 2 mm brain slices revealed no accumulation of F8-SIP-Alexa-750 in non-tumor areas resulting in tumor-specific visualization of experimental tumors (*n* = 6 per group, representative animals are shown in Figure 5A, 5C and 5E and Supplementary Figure 1). Accumulation in the brain was not observed if the HyHEL-Alexa-750 control was injected (*n* = 3, NaCl treatment, Figure 5E–5F). MRI imaging was applied with contrast-enhanced T_1_-weighted MRI and in T_2_-weighted MRI to visualize edema (Figure 5B, 5D, 5F). There was a significantly lower MRI tumor volume (Figure 6A, median and [IQR]: 33.8 [2.9 46.4] vs. 106.2 [43.6 129.6], Mann-Whitney *U* test, *p* = 0.026) and F8-SIP-Alexa-750 positive NIRF volume (Figure 6B, median and [IQR]: 38.7 [0.7 62.1] vs. 83.5 [51.2 108.8], Mann–Whitney *U* test, *p* = 0.041) in sunitinib treated animals compared to NaCl control as analyzed by semi-automated WEKA segmentation (see methods and Supplementary Figure 1B) in *ex vivo* brain slices. Analyzing *ex vivo* NIRF images of brain slices, the ratio of fluorescence of the ipsilateral tumor bearing hemisphere and the contralateral hemisphere was higher in NaCl treated group though this difference did not reach significance (Figure 6C, two tailed Mann–Whitney *U* test, *p* = 0.132 and Supplementary Figure 1B). Analyzing only the NIRF image areas from the WEKA segmentation (Figure 6D and Supplementary Figure 1B) showed that the range of targeted ratio values in sunitinib treated animals encompassed the range of ratio values in the NaCl treated group. In summary, MRI tumor volume and angiogenic volume as analyzed by NIRF imaging positive volume from brain slices positively correlates in the NaCl treated group but showed no significant difference between groups (overall positive regression with Spearman r of 0.559 with *p* = 0.063, *n* = 12).

### Molecular imaging of therapy resistant glioma vessels

As shown by IVFM, vascular accumulation of F8-SIP-Alexa-555 was significantly higher in treated, remaining tumor vasculature as compared to untreated tumor vasculature (Figure 3). NIRF imaging revealed that F8-SIP-Alexa-750 accumulation was specific and HyHEL-Alexa-750 control did not show accumulation in the brain of tumor bearing mice (Figure 5A and 5E). In parallel, NIRF imaging of sliced brain tissue revealed higher maximum NIRF signals in the treated group compared to control (Figure 5A and 5C). To quantify this, we calculated NIRF ratios of the ipsilateral (tumor bearing) hemisphere versus the contralateral hemisphere of corresponding brain slices (see Supplementary Figure 1), which showed lower but not significantly different F8 SIP ratios in the sunitinib treated group compared to controls (median and [IQR]: 2.8, [1.9 4.4] versus 1.8 [1.0 2.9], *p* = 0.132, Figure 6C). In addition, targeted ratios were calculated after automated segmentation into tumor and non-tumor areas (with ROIs corresponding to NIRF positive area only instead of entire hemisphere, see Supplementary Figure 1B), there was a wide spread of ratio values and no significant difference between treatment groups (Figure 6D, *p* = 0.310, Mann-Whitney-*U*-test). In summary, NIRF imaging demonstrated comparable fluorescence signal within therapy resistant tumor areas compared to untreated tumor areas despite lower vessel density in histological analysis.

## DISCUSSION

Our study provides detailed characterization of the F8-SIP microvascular binding process and preferential binding sites during glioma angiogenesis and after antiangiogenic treatment. We demonstrate that F8-SIP binds specifically to tumor vessels in a time and blood flow dependent manner. After antiangiogenic treatment, F8-SIP accumulates in therapy-resistant tumor vessels enabling molecular based visualization and targeting of therapy resistant tumor vessels.

In order to improve selection of GBM patients that benefit from antiangiogenic treatment und to select patients that are resistant to treatment, specific visualization of tumor vasculature is critical. In this regard there is a lack of specific tumor vasculature imaging in clinical practice [6]. F8-SIP was found to specifically target glioma vasculature, but the microvascular distribution especially during antiangiogenic intervention has not been investigated so far [7]. In our experiments, IVFM showed rapid attachment of the F8 antibody to glioma vasculature within 2 h and stable binding for at least 24 h. NIRF imaging underlines tumor specificity of F8-SIP in comparison with a non-specific HyHEL-Alexa-750 labeled probe in an orthotopic glioma model and showed a close correlation between measured F8-SIP-Alexa-750 fluorescence signal intensities and tumor volumes measured with MRI.

In a secondary process, extravasation of F8-SIP-Alexa-555 was detected at a lower level as compared to vascular accumulation showing the optimal signal-to-background ratio 4 h after injection which remained stable for 24 h. Therefore, F8-SIP may be suitable for therapeutic or diagnostic vascular targeting strategies over a time period of at least 24 h. In comparison to microvascular distribution of L19-SIP, which targets the extradomain B of fibronectin, F8-SIP shows a superior signal-to-background ratio and maintains it over a prolonged time period making it a suitable tool for clinical applications e.g. after radiolabeling [8].

Extravasation of F8-SIP may be influenced by many factors including tumor specific characteristics like microvascular permeability, interstitial diffusion coefficient and expression of EDA in the interstitium [5, 7, 9]. Interestingly, the F8-SIP antibody demonstrated preferential binding to immature vessels like angiogenic sprouts – where alternatively spliced fibronectin is expressed predominantly due to massive vascular remodeling – and to high flow blood vessels, postulating a hemodynamic dependency of the binding process and a preference for immature vessels [10, 11]. Tumor vessels that are resistant to antiangiogenic treatment are characterized by altered microvascular hemodynamics and different pericyte-endothelial cell interactions as compared to normal tumor vasculature [12]. F8-SIP-Alexa-555 showed significantly increased accumulation in sunitinib-resistant tumor vessels as compared to normal tumor vasculature. In these resistant tumor vessels, accumulation of F8-SIP-Alexa-555 also occurred on the ab-luminal side of the tumor vessel verifying that location of the molecular target was not changed in response to sunitinib treatment. NIRF image analysis further underlined significantly increased binding of F8-SIP-Alexa-750 in resistant tumor vasculature in an orthotopic model, pointing out that the tumor microenvironment (orthotopic vs. subcutaneous tumor model) did not influence this effect. It may be possible, that tumor vessel specific characteristics are rather responsible for increased accumulation in sunitinib-resistant tumor vessels than for interactions with the tumor microenvironment. One important reason may be found in increased microvascular blood flow rate in therapy resistant tumor vessels. It was demonstrated in an experimental setting investigating the effects of altered micro-hemodynamics in response to antiangiogenic treatment, that microvascular delivery of chemotherapy is increased with improved microvascular blood flow rate [12]. Consequently, it must be supposed that delivery of F8-SIP is increased in resistant tumor vessels and therefore increased microvascular binding occurs. Nevertheless, wall shear stress and pericyte-endothelial interactions may also be involved in this process. However, as shown in previous studies Sunitinib does not lead to significant changes of microvascular permeability or wall shear stress [12, 13]. Interestingly, another antiangiogenic agent bevacizumab resulted also in significant microvascular alterations such as reduction of microvascular density, vascular volume and interstitial pressure, however without concurrent alterations in the uptake of radioactive tracers in tumors [14].

In NIRF image analysis, F8-SIP-Alexa-750 fluorescence was non-significantly reduced in response to antiangiogenic treatment. This can be explained by the heterogeneity observed in treated tumors with tumor areas that respond well to antiangiogenic treatment showing no or very low accumulation of F8-SIP-Alexa-750 while resistant areas are characterized by increased accumulation. This phenomenon is visualized by therapy-resistant hot-spots within the tumor area that may be differentiated from areas that are responding to the antiangiogenic compound. Translation of this technique into clinical application may enable identification of treatment responders and patients that are resistant to antiangiogenic treatment. Moreover, this phenomenon may allow specific therapeutic targeting of resistant tumor vessels in order to improve efficacy of antiangiogenic but also other anti-glioma compounds.

## MATERIALS AND METHODS

### Tumor cells

Human SF126 glioma cells (#IFO50286, JCRB cell bank, Japan) were routinely maintained at 37° C in 5% CO_2_ humidified incubators in DMEM with 4.5 g/L glucose supplemented with 10% fetal bovine serum (PAA GmbH, Linz, Austria).

### Bio-distribution analysis with intravital fluorescence microscopic imaging

All experiments were approved by Landesamt für Gesundheit und Soziales Berlin (G0003/08, G0200/07) and were carried out according to the guidelines for animal care and experimentation.

For bio-distribution analysis 3 × 10^5^ SF126 glioma cells were implanted into dorsal skinfold chambers in nude mice (*n* = 23 and 7 controls) after 2 days. Intravital fluorescence microscopy (IVFM) analysis was successfully performed on day 7 after SF126 glioma cell implantation in 5 mice per group. Two animals were excluded due to diminished DSC quality in the controls and 3 animals in the glioma group. Microscopy was performed during injection of F8-SIP-Alexa-555 (t = 0) as well as 4 h (t = 4 h) and 24 h (t = 24 h) after intravenous application. F8 small immunoprotein was labeled with Alexa-555 for the bio-distribution analysis in the dorsal skinfold chamber experiments. The microsurgical techniques for the implantation of tumor cells into the dorsal skinfold chamber has been described previously [15]. Mice were anesthetized by intraperitoneal injection with a mixture of ketamine (100 mg/kg) and xylazine (10 mg/kg). Intravital fluorescence video microscopy (IFVM) was performed by epi-illumination techniques using a modified Axiotech vario microscope (Attoarc; Zeiss, Jena, Germany). Microscopic images were recorded through a charge-coupled device (CCD) video camera with an optional image intensifier for weak fluorescence (Kappa, Gleichen, Germany) and transferred to a S-VHS video system (Panasonic) for offline analysis. Offline analysis was performed using a computer assisted analysis system (CAPIMAGE; Zeintl Software Engineering, Heidelberg, Germany). Microvessels were visualized by contrast enhancement with 2% FITC-conjugated dextran (0.1 ml, intravenous; molecular weight 150,000 Dalton; Sigma). Simultaneous *in vivo* application of the Alexa 555-labeled F8-SIP and the use of green-light epi-illumination allowed for sequential analysis of bio-distribution. The detailed bio-distribution analysis is described in supplementary section.

### Antiangogenic therapy

Treatment with sunitinib was initiated 7 days after SF 126 glioma cell implantation. All animals in the treatment group (*n* = 9 total) received daily intraperitoneal injections of sunitinib (40 mg/kg/day) and 6 of them finished treatment over 6 days. Two of these animals had to be excluded due to diminished DSC quality. For analysis of tumor micro-hemodynamics we assessed microvascular red blood cell velocity (RBCV; mm/s), total and functional vessel density (TVD, FVD; cm/cm^2^), perfusion index (%), vascular diameter (D, mm), microvascular permeability (P; the ratio between intra- and extravascular contrast) and microvascular blood flow rate (Q_*v*_; nL/s) on day 14 in 1-3 tumor fields (colour coded per animal in Figure 3B–3F) as described previously [16, 17].

### Stereotactic tumor cell implantation

For NIRF image analysis and small animal magnetic resonance imaging (MRI), an orthotopic tumor model was used. A total of *n* = 38 eight-week old male nude mice (Charles River, Wilmington, Massachusetts, USA) were used for the experiments. In brief, the left temporalis muscle was detached from the scull and a small whole was drilled in the middle of a virtual line between the lateral corner of the left eye and the ear. Using a Hamilton syringe (7001 N, ga 0.47/70 mm/pst3, Hamilton, Bonaduz, Switzerland) 20000 SF126 cells in a volume of 1µl were inoculated stereotactically at a depth of 1.7 µm into the left striatum. Bupivacain gel was applied topically for local pain relief and skin wounds were closed using tissue glue.

### Randomization, concealment of treatment allocation, blinding of treatment with sunitinib, and exclusion criteria

MRI was performed on day 4 after inoculation of SF126 cells. One animal out of the initial 38 orthotopic tumor model mice was excluded because the tumor was 10 times bigger than the average of all other tumors prior randomization to treatments after the first MRI scan.

Subsequently, mice were randomized semi-automatedly to treatment (www.graphpad.com/quickcalcs/randomize1.cfm) within strata of tumor volume to body weight ratios aiming for an equal distribution of this virtual measure in treatment groups as indicated below. For antiangiogenic experiments, treatment with sunitinib was started on day 4 after orthothopic tumor inoculation and performed either for

(i) 5 days with a daily dose of 40 mg/kg bodyweight (BW) : *n* = 4, or

(ii) 5 days with a daily dose of 80 mg/kg BW: *n* = 4, or

(iii) on day 4 and day 7 in a dose of 80 mg/ kg BW: *n* = 12.

(iv) Controls received daily injections of 0.95% Natrium chloride (NaCl): *n* = 17, of which 3 mice additionally got injected with HyHEL-Alexa-750 as control marker.

Treatment allocation was blinded to all researchers that performed MRI analysis, NIRF imaging or histology throughout the experiment.

### Near infrared fluorescence imaging

#### Fluorescence reflectance imaging

Fluorescence epi-illumination images were acquired with a system described in detail in [18]. Light from a TEC-cooled, fiber-coupled laser diode emitting at 670 nm (1305-9MMF-67010 Intense-US, Laser 2000, Wessling, Germany) was coupled to an optical switch (LightTech Fiberoptics Inc, San Leandro, CA) and directed further into a dark chamber, where two overlapping light cones (about 4 cm in diameter) illuminated the object from above. A back-illuminated nitrogen-cooled CCD camera (Vers Array 512, 512 × 512 px, Roper Scientific Inc., Duluth, GA) equipped with a focusing lens system (Nikkor macro lens, f = 50 mm, f/1.2, Nikon, Duesseldorf, Germany) and two 780-nm interference filters (FWHM 20 nm, Andover Corp., Salem, NH) detected the emitted fluorescence light. Depending on the fluorescence’s intensity, data acquisition times varied between 60 and 120 s.

Mice were injected F8-SIP-Alexa750 targeting the extradomain A of fibronectin or HyHEL-Alexa-750 control antibody targeting hen egg lysozyme with irrelevant specificity for mice (HH10-SIP (HyHEL)- expression, purification is described in supplemental section in detail). NIRF imaging was started 24 h after injection. Standard curves of freshly thawed aliquots with three serial 10 fold dilutions of 1 µl of antibody or PBS control were used to display fluorescence label adjacent to the NIRF of mice, brains, or slices. Non-invasive NIRF imaging was analyzed qualitatively. NIRF imaging-signal analysis and WEKA segmentation is described in supplemental section.

### Small animal magnetic resonance imaging

Magnetic resonance imaging (MRI) was performed on days 4 and 9 after tumor cell implantation using a 7 Tesla rodent scanner Pharmascan 70/16AS (Bruker, BioSpin, Germany) and a 1-H-RF-quadrature volume resonator with an inner diameter of 20 mm (Rapid Biomed). During the examinations mice were placed on a heated circulating water blanket to ensure a constant body temperature of 37° C. Anesthesia was maintained with 1.5–2.0% isoflurane (Forene, Abbot, Wiesbaden, Germany) delivered in a O_2_/N_2_O gas mixture (0.3/0.7 l/min) via a face mask under constant ventilation monitoring (Small Animal Monitoring & Gating System, SA Instruments, Stony Brook, New York, USA). For imaging the mouse brain contrast-enhanced T_1_- and T_2_-weighted 2D TurboRARE spin-echo sequences were used (imaging parameters: for T_1_: TR/TE = 800/10.6 ms, RARE factor 2, 4 averages and for T_2_: TR/TE = 4200/36 ms, RARE factor 8, 4 averages). For each contrast 20 axial slices with a slice thickness of 0.5 mm, a field of view of 2.60 × 2.60 cm and a matrix of 256 × 256, resulting in an in-plane resolution of 102 µm × 102µm, were positioned over the brain. For contrast-enhanced T_1_-weighted MRI 100 µl Gd-DTPA (Magnevist; Bayer, Leverkusen; Germany) 0.5 mmol/kg was injected intravenously. Tumor volumes were determined by drawing volumes of interests with Analyze 10.0 (Biomedical Imaging Resource, Rochester MN).

### Immunohistochemistry

Acetone-fixed cryo-sections (5µm) were used for immunohistochemistry after bio-distribution analysis of Alexa 555-labelled F8-SIP. Co-localization of Alexa555-labelled F8-SIP and endothelial cells by a rat monoclonal CD31 antibody MEC13.3 (BD Pharmingen, Heidelberg, Germany) was visualized by an Axioplan2 microscope (Zeiss, Jena, Germany) and representative fluorescence images are shown (Figure 1B, 1C).

Mice of the NIRF imaging group were transcardially perfused with 20 ml of 0.9% NaCl solution and subsequently with 4% paraformaldehyde (PFA) in 1× PBS immediately after the MRI postscan. The organs were postfixated in 4% PFA at 4° C overnight, washed twice in 1× PBS and then dehydrated in 30% sucrose in 1 × PBS. The tissue was embedded in paraffin and sectioned (4 µm). The sections were incubated in xylol for 3 × 5 min and a descending sequence of ethanol concentrations (100%, 96%, 80%, 70%, 1 min each) to remove the paraffin from the tissue. This was followed by antigen retrieval in 10 mM citrate buffer pH 6,0. We performed a double fluorescence immunostaining with a rat monoclonal CD31 antibody (PECAM-1; Dianova, DIA 310, Dianova, Hamburg, Germany) for endothelial cell visualization and an anti-Ki67 antibody (ThermoScientific, #RM-9106-S-Fluoreszenz, USA) for endothel cell proliferation. Sections were mounted with immunoselect antifading mounting medium containing DAPI (Dianova, Hamburg, Germany), for staining of the nuclei. Quantification of the vessel density and endothelial cell proliferation (CD31 and Ki67 positive endothelial cells) were performed using the average of 2 sections per tumor and 5–6 tumor fields derived thereof in 20 fold magnification by open source Java image processing Image J (NIH Image, Rockville, USA). Data points in Figure 4E–4F represent average values within each animal (*n* = 5 per group).

### Statistical analysis

Quantitative data are given as mean and 95% confidence interval (Figure 2A–2B) or median with limits of the interquartile range [IQR, 25th and 75th percentile] and illustrated in scatter and dot plots with individual data points for each group or animal, respectively. Nested data within a given animal are color coded where appropriate (tumor fields in Figure 3). Boxplots represent median as well as lower and upper limit of the interquartile range, with whiskers giving the minimum and maximum values. For analysis of differences between two groups Mann-Whitney-*U* test or Wilcoxon test were applied. Two-way repeated measures ANOVA with Sidak’s multiple comparison tests were applied for data in Figure 2A–2B. Three factorial Kruskal-Wallis ANOVA on ranks, followed by Dunn’s posthoc test with NaCl as reference was applied for Figure 4A–4B and mean differences with 95% confidence intervals are given.

Statistical analysis was performed using Prism 6.07 (GraphPad, San Diego, USA). All tests were two-sided and *p* < 0.05 was considered statistically significant. No further adjustment for the overall number of statistical tests was performed in this exploratory study.

## SUPPLEMENTARY MATERIALS FIGURE



## Abbreviations

BW: Bodyweight
CCD: charge-coupled device
D: vascular diameter
EDA: Extradomain A
FVD: functional vessel density
GBM: glioblastoma multiforme
IVFM: Intravital fluorescence microscopy
MRI: magnet resonance imaging
NaCL: Natrium chloride
NIRF: near infrared fluorescence
P: microvascular permeability
PFA: paraformaldehyde
Q_*v*_: microvascular blood flow rate
RBCV: red blood cell velocity
SIP: small immunoprotein
TVD: total vessel density
