# Supplementary material for: Targeting the extradomain A of fibronectin allows identification of vascular resistance to antiangiogenic therapy in experimental glioma
Source: Oncotarget. 2018 Jun 12;9(45):27760–72. doi: 10.18632/oncotarget.25570 (PMC6021244; doi:10.18632/oncotarget.25570)
Supplement: Supplementary file 1 [file oncotarget-09-27760-s001.pdf]

Targeting the extradomain A of fibronectin allows identification of vascular resistance to antiangiogenic therapy in experimental glioma

SUPPLEMENTARY MATERIALS

Bio-distribution analysis

Bio-distribution was studied by measuring fluorescence intensity in the vascular and interstitial compartment in a total number of 6-9 tumor vessels per animal (Figures 1, 2). For the measurement of fluorescence intensity we defined 2 regions of interest (area: 250–500 µm²) per analyzed tumor vessel: (i) the vascular compartment referring the vascular wall and the close perivascular area (defined as an area of 250–500 µm² with a width of 3 µm located next to the analyzed tumor vessel) and (ii) the interstitial compartment (area of 250–500 µm²) located in the interstitial space with a distance of 20 µm from the analyzed tumor vessel. Before analysis, we measured and subtracted interstitial fluorescence intensity in order to exclude auto fluorescent activity.

NIRF imaging-signal analysis and WEKA segmentation

All image processing steps were performed within the program suite Fiji [1] or Matlab R2007b (The MathsWorks Inc.). 32 bit images of five brain slices per animal were background-subtracted (250 pxl) using a rolling ball algorithm and filtered (6 pxl). Images were used to train the automated WEKA segmentation tool with three signal-positive and three signal negative areas per image [2]. By applying the generated WEKA classifier, the median ipsi- and contralateral NIRF-intensities of selected NIRF-positive areas on 2 mm thick brain sections were measured and the ratios calculated.

Moreover, NIRF signal positive “tumor volumes” were calculated by summing up all NIRF-positive pixel (resolution: 98 µm × 98 µm) over all corresponding brain slices multiplied by the slice thickness of 2 mm (Supplementary Figure 1A–1B).

HH10-SIP (HyHEL)- expression, purification

HH10-SIP-expressing cells were grown in Dulbecco’s modified Eagle medium (DMEM, Invitrogen, Darmstadt, Germany) supplemented with 10% FCS-Gold and 1% penicillin / streptomycin (both PAA Laboratories, Pasching, Germany). The supernatants were collected and cell debris removed by centrifugation at 1.200 × g, 20 min. 4° C. Affinity beads were generated by coupling hen egg lysozyme to cyano bromide sepharose (both Sigma-Aldrich, Steinheim, Germany). The secreted HH10-SIP protein was passed over a G25 sepharose (GE Healthcare, Buckinghamshire, UK) to reduce unspecific binding to sepharose beads. Binding to henn-egg lysozyme was carried out for 10 h at 4° C on a rotator. The solution was transferred to a gravity flow column and bound protein was washed with 10 column volumes of 0.1 M NaCl in PBS, pH 7.4, 0.5 mM EDTA once with and subsequently without 0.1% Tween. Elution was performed with 100 mM triethylamine (Sigma-Aldrich, Steinheim, Germany) in PBS. The fractions were neutralized immediately by addition of 100 mM Tris buffer, pH 8.0. HH10-SIP containing fractions were pooled, re-buffered in 100 mM sodium bicarbonate buffer, pH 8.3 and concentrated to 3 mg/ml in Amicon Ultra-4, 3,000 NMWL centrifugal filter devices (Millipore, Cork, Ireland).

Alexa Fluor 750 carboxylic acid succinimidyl ester (Invitrogen, Darmstadt, Germany) was added at 20 molar excess to the protein solution and coupled for 1 h at ambient temperature and subsequently for 10 h at 4° C. Excess fluorescent dye was removed by gel filtration chromatography over a PD-10 column (GE Healthcare, Buckinghamshire, UK) equilibrated with PBS. The resulting Alexa 750-labeled HH10-SIP with a degree of labeling of 5 according to the UV spectra was concentrated to 1.00 mg/ml and stored in aliquots at −80° C.

REFERENCES

1. Schindelin J, Arganda-Carreras I, Frise E, Kaynig V, Longair M, Pietzsch T, Preibisch S, Rueden C, Saalfeld S, Schmid B, Tinevez JY, White DJ, Hartenstein V, et al. Fiji: an open-source platform for biological-image analysis. Nat Methods. 2012; 9:676–82. https://doi.org/10.1038/nmeth.2019.
2. Arganda-Carreras I, Kaynig V, Rueden C, Eliceiri KW, Schindelin J, Cardona A, Sebastian Seung H. Trainable Weka Segmentation: a machine learning tool for microscopy pixel classification. Bioinformatics. 2017; 33:2424–6. https://doi.org/10.1093/bioinformatics/btx180.

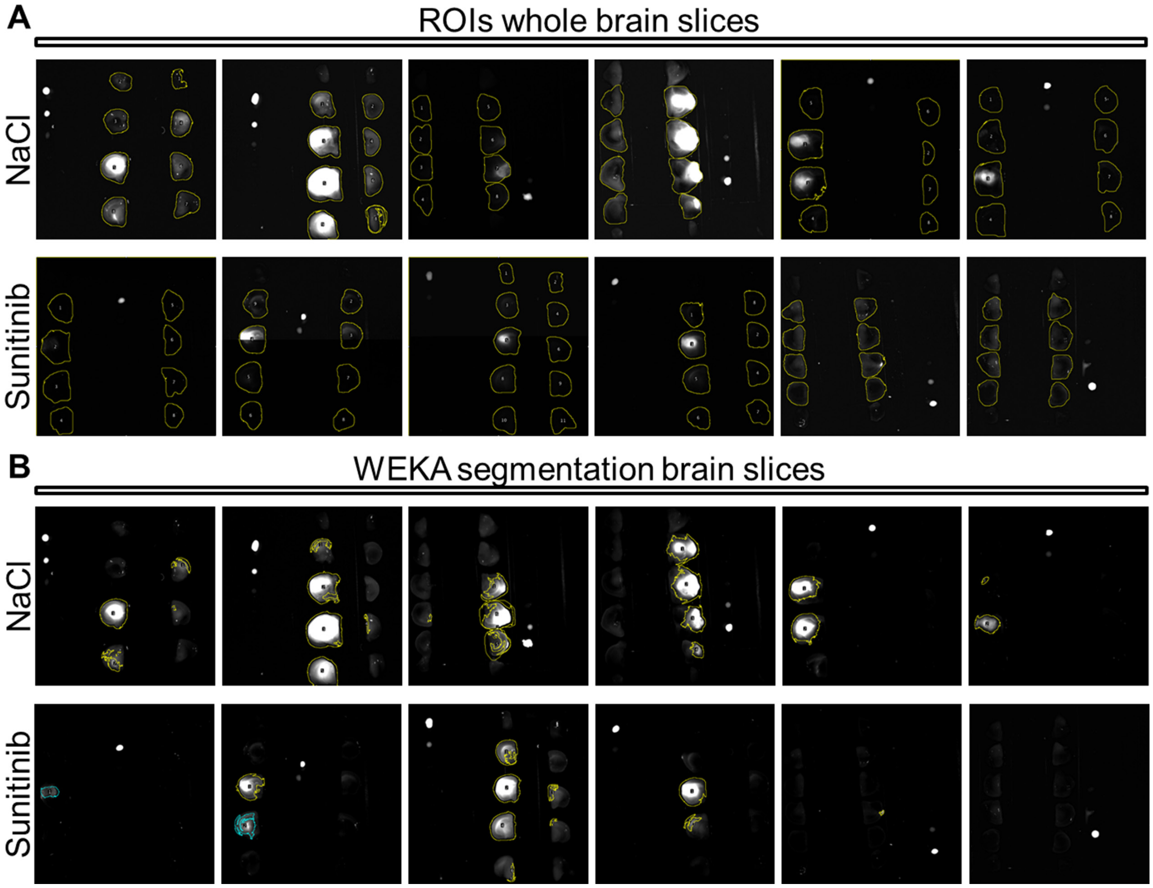

Supplementary Figure 1: (**A** and **B**) Brains were cut into 2 mm thick slices and NIRF imaging was performed using F8-SIP-Alexa-750 (6 animals per group, quantitative analysis Figure 6B–6D). Whole brain slice volume is demonstrated for all NaCl treated glioma (upper panel) or sunitinib treated glioma (lower panel). (B) Angiogenic NIRF volumes after sunitinib treatment based on the F8-SIP antibody distribution using WEKA segmentation. NIRF signal positive "tumor volumes" were calculated by summing up all NIRF-positive pixel (resolution: 98 µm × 98 µm) over all corresponding brain slices multiplied by the slice thickness of 2 mm
